# Lpar2b Controls Lateral Line Tissue Size by Regulating Yap1 Activity in Zebrafish

**DOI:** 10.3389/fnmol.2018.00034

**Published:** 2018-02-09

**Authors:** Xueqian Wang, Haitao Hou, Kaida Song, Zhiqiang Zhang, Shuqiang Zhang, Ying Cao, Liming Chen, Qing Sang, Fang Lin, Hui Xu

**Affiliations:** ^1^Key Laboratory of Neuroregeneration of Jiangsu and Ministry of Education, Co-innovation Center of Neuroregeneration, Nantong University, Nantong, China; ^2^State Key Laboratory of Genetic Engineering, Institutes of Biomedical Sciences, Fudan University, Shanghai, China; ^3^School of Life Science and Technology, Tongji University, Shanghai, China; ^4^Biochemistry and Biological Product Institute, School of Life Sciences, Nanjing Normal University, Nanjing, China; ^5^MOE Key Laboratory of Contemporary Anthropology and School of Life Sciences, Fudan University, Shanghai, China; ^6^Department of Anatomy and Cell Biology, Carver College of Medicine, University of Iowa, Iowa City, IA, United States

**Keywords:** LPA, organ size, lateral line, neuromast, hair cells, zebrafish

## Abstract

LPA signaling plays important roles during cell migration and proliferation in normal and pathological conditions. However, its role during sensory organ development remains unknown. Here we show a LPA receptor Lpar2b is expressed in the posterior lateral line primordium (pLLP) and mechanosensory organs called neuromasts (NMs) in zebrafish embryos. Lpar2b loss-of-function significantly reduces the number of NMs and hair cells in the posterior lateral line (pLL). Further analysis reveals that Lpar2b regulates the patterning and tissue size of the pLLP. Interestingly, we show that knocking down a Hippo effector Yap1 phenocopies the result of Lpar2b depletion, and Lpar2b regulates the phosphorylation and activity of Yap1 in the pLLP. Importantly, a phosphorylation-resistant Yap1 rescues pLLP size and NM number in Lpar2b-depleted embryos. Our results indicate Lpar2b controls primordium size and NM number by regulating Yap1 activity in the lateral line system.

## Introduction

The morphogenesis and size control of organs are important features of metazoans and play critical roles in animal development and regeneration (Tumaneng et al., 2012). Abnormal organ size contributes to many pathological conditions in humans, including hypertrophy and cancer (Yu et al., 2015; Ikeda and Sadoshima, 2016). During development, the control of final organ size is determined by the precise regulation of cell number and cell size. In general, cell number is affected by both cell proliferation and cell death, while cell size is dependent on cell growth. Major signaling pathways regulating organ size include the Hippo and mTOR pathways (Penzo-Méndez and Stanger, 2015). It has been shown that the Hippo pathway mainly regulates cell number, while mTOR is primarily responsible for cell growth (Yu et al., 2015; Saxton and Sabatini, 2017). Initially identified in *Drosophila*, the Hippo pathway is an important regulator of cell proliferation and cell death. The core components of this pathway consist of Mst1/2, Sav, Lats1/2, and Mob1, and key downstream effectors YAP/TAZ that function as transcription co-activators (Tumaneng et al., 2012). Upon Hippo activation, Mst1/2 interacts with Sav to phosphorylate the Lats1/2-Mob1 complex, which in turn phosphorylates YAP and TAZ, causing their cytoplasmic retention and inactivation (Camargo et al., 2007; Zhao et al., 2007, 2010). Mutation of the components of the Hippo pathway resulted in tissue overgrowth phenotypes (Zhou et al., 2009; Halder and Johnson, 2011), highlighting the role of this pathway in organ size control.

The zebrafish posterior lateral line (pLL) is an excellent system to study tissue morphogenesis and organ size control, due to its simplicity in structure and accessibility for *in vivo* imaging (Wada and Kawakami, 2015). The lateral line is a sensory system in fish and amphibians to detect water movement and consists of mechanosensory organs called neuromasts (NMs) under their skin (Xu et al., 2014). During zebrafish development, the pLL is generated by the posterior lateral line primordium (pLLP), a group of about 100 cells collectively migrating from the otic vesicle to the tip of the tail along the myoseptum (Dalle Nogare and Chitnis, 2017). The pLLP is morphologically patterned along its direction of migration into two regions, the leading zone and the trailing zone. Cells in the leading zone display mesenchymal characteristics and actively extend protrusions responding to external guidance cues (Xu et al., 2014). In contrast, cells in the trailing zone undergo a tissue rearrangement through “pseudo” mesenchymal-epithelial transition (MET) and form rosette-like structures called proneuromasts (pro-NMs; Thomas et al., 2015). Pro-NMs then periodically separate from the primordium and are deposited along the path of migration to develop into mature NMs that contain mechanosensory hair cells at their center (Nechiporuk and Raible, 2008). It has been shown that FGF/Notch signaling drives the formation of apical constrictions and rosette structures in the trailing zone (Dalle Nogare and Chitnis, 2017), and periodic NM deposition is also influenced by Wnt/Fgf-dependent cell proliferation in the primordium (Aman et al., 2011). However, precise mechanisms underlying pro-NM separation and deposition remain elusive.

Lysophosphatidic acid (LPA) is a bioactive phospholipid best known for its ability to stimulate cell proliferation, migration and survival both in normal and malignant condition (Sheng et al., 2015). LPA is mainly produced by an enzyme autotaxin (ATX, gene name *Enpp2*) which converts lysophosphatidylcholine (LPC) to LPA (Tokumura et al., 2002; Umezu-Goto et al., 2002). To date, six LPA receptors LPA1-LPA6 (human gene name *LPAR1-LPAR6, Lpar1-Lpar6* in mice and *lpar1-lpar6* in zebrafish) have been identified and characterized (Riaz et al., 2016). LPA receptors are seven-transmembrane GPCRs that bind LPA with varying affinities and signaling through specific heterotrimeric G proteins (Sheng et al., 2015; Riaz et al., 2016). Based on their structures, LPA receptors are divided into two subgroups. LPA1-3 belong to the endothelial differentiation gene (EDG) family and show high sequence homology to the S1P receptors (Hecht et al., 1996; An et al., 1998; Bandoh et al., 1999), while LPA4-6 are more closely related to the purinergic receptors (Ishii et al., 2009). LPA signaling play important roles in vascular development (Tanaka et al., 2006), nervous system function (Fukushima et al., 2002; Matas-Rico et al., 2008), lymphocyte homing (Kanda et al., 2008) and tumor progression (Houben and Moolenaar, 2011). *Lpar1*-null mice display 50% perinatal lethality and olfactory defects (Contos et al., 2000). Interestingly, these mice are growth-restricted and appear smaller in size compared with wild-type siblings (Hecht et al., 1996), indicating a role of the LPA signaling in animal growth. It was shown that LPA receptors are the upstream regulator of the Hippo pathway in cell culture systems (Yu et al., 2012). Specifically, G_12/13_-coupled LPA receptors inhibit the Hippo pathway kinases Lats1/2 by an unknown mechanism, thereby activating YAP and TAZ (Yu et al., 2012). However, it's not clear if such regulation plays a role in organ development and size control *in vivo*.

Although LPA signaling has been studied previously in other systems, its role in the development of the sensory system remains unknown. Here we show that a LPA receptor Lpar2b is expressed in the migrating pLLP and deposited NMs in zebrafish. Morpholino (MO)-mediated Lpar2b depletion resulted in a significantly decreased number of NMs and hair cells. Surprisingly, we found that this phenotype was not caused by abnormal pLLP migration or pro-NM morphogenesis, but by reduced tissue size of the pLLP. Interestingly, the NMs phenotype in Lpar2b morphants can be recapitulated by knocking down a Hippo pathway effector Yap1. Finally, we show that Lpar2b controls pLLP size and NM number by regulating Yap1 phosphorylation. Taken together, our study revealed a novel role of LPA-Hippo signaling in organ size control in the lateral line system and suggests this function may also apply to the development of other organs.

## Materials and methods

### Ethics statement

All animals used in this study were treated in accordance with the NIH guidelines for the Care and Use of Laboratory Animals (https://oacu.oir.nih.gov/regulations-standards). Animal experiments were ethically approved by the Administration Committee of Experimental Animals, Jiangsu, China (Approval ID Number: SYXK(SU)2007-0021).

### Zebrafish maintenance and manipulation

Wild-type (AB), transgenic *Tg(-8.0cldnb:lynEGFP) (Haas and Gilmour*, *2006**), Tg(Brn3c:mGFP)* (Xiao et al., 2005), *Et(gata2:EGFP)*^*mp*189*b*^ (Sang et al., 2014) zebrafish were used in this study. Zebrafish embryos were obtained through natural mating and maintained at 28°C in the fish facility. Embryo was treated with 0.2 mM 1-phenyl-2-thiourea (PTU) to inhibit pigment development. When developed to desired stages as previously described (Kimmel et al., 1995), embryos were collected and fixed with 4% paraformaldehyde (PFA) in phosphate-buffered saline (PBS) overnight at 4°C.

### Whole-mount *in situ* hybridization

Sense and anti-sense RNA probes labeled with digoxigenin (DIG) were synthesized according to manufacturer's protocol with the DIG RNA Labeling Kit (SP6/T7) (Roche Applied Science). Whole-mount *in situ* hybridization was performed as previously described (Thisse and Thisse, 2008).

### Morpholino and mRNA injection

Morpholinos synthesized by Gene Tools LLC were injected into zebrafish embryos at 1–2 cell stage. MOs were prepared at a stock concentration of 1 mM and working concentration of 0.3 mM unless otherwise stated. A standard Control MO (Con-MO) was used as a control. The sequences of MOs targeting LPA receptors in the study: Lpar2b MO (translation-blocking MO) 5′-TCTGATTGGCTGAGCTGAAGGGATC-3′, Lpar2b MO2 (splicing MO) 5′-AGACACACTCAGAGTCGTACCTGCC-3′. Lpar2b MO was efficient to suppress the protein translation of the GFP-tagged target transcript (Supplementary Figures 1A,B). The efficacy of the splicing MO targeting Lpar2b (Lpar2b MO2) was also confirmed by RT-PCR (Supplementary Figure 1E). The Yap1 MO (translation-blocking, 5′-CTCTTCTTTCTAT CCAACAGAAACC-3′) has been previously described and validated (He et al., 2015).

The constitutively active Yap1 (caYap1) was constructed by amino acid substitutions (S87A and S335A) of wild-type zebrafish Yap1 as described previously (Zhao et al., 2007). 5′-capped mRNA was synthesized using the mMESSAGE mMACHINE® Kit (Thermo Fisher Scientific) and 100–200 pg of the purified mRNA was injected into one-cell stage zebrafish embryos.

### Guide-RNA (gRNA) and Cas9 mRNA synthesis and microinjection

The Cas9 mRNA was generated by *in vitro* transcription with a linearized plasmid pXT7-Cas9 as previously described (Gong et al., 2017). The *lpar2b* gRNA was transcribed from the DNA template of amplified PCR products of the pT7 plasmid with a specific forward primer 5′-TAATACGACTCACTATAagaacatcagcgatacgtggGTTTTAGAGCTAGAAATAGC-3′, and a universal reverse primer 5′-AAAAAAAGCACCGACTCGGTGCCAC-3′ (Chang et al., 2013). Two hundred and fifty pg Cas9 mRNA and 15 pg *lpar2b* gRNA were injected into one-cell stage transgenic *Et(gata2:EGFP)*^*mp*189*b*^ embryos. At 24 hpf, injected embryos were randomly collected and genomic DNA were extracted to determine the indel mutations by DNA sequencing.

### BrdU incorporation and whole-mount immunofluorescence

At 32 hpf, embryos were incubated in 10 mM BrdU 15% DMSO in fish water on ice for 30 min and then recovered at 28.5°C for 1 h. Embryos were then fixed in 4% PFA at 4°C overnight. Whole-mount immunofluorescence was performed as previously described (He et al., 2016). The following primary antibodies were used: BrdU (1:200, Abcam), GFP (1:200, Invitrogen), p-Yap1 (1:200, Cell Signaling Technology). The BrdU index is calculated as (number of BrdU+ cells in pLLP)/(total number of pLLP cells).

### Tunel assay

Transgenic *Tg(-8.0cldnb:lynEGFP)* embryos developed to 30–32 hpf were fixed in 4% PFA at 4°C overnight. TUNEL assay was performed using a kit as previously described (ApopTag Red *in situ* Apoptosis Detection kit, Millipore; Xu et al., 2014). The presence of TUNEL+ cells (red fluorescence in the pLLP) was analyzed under a fluorescence microscope.

### Microscopy, time-lapse imaging, and analysis

Embryos were mounted and photographed using a Leica DMI6000 inverted microscope or a Leica M165FC stereofluorescence microscope. For *in vivo* time-lapse imaging, live embryos were anesthetized in 0.01% tricaine and mounted in 1% low melting-point agarose. Fluorescence time-lapse images were taken on a Leica DMI6000 microscope or a Zeiss LSM700 confocal microscope (Carl Zeiss, Inc.). To assess the rosette structures, 4–5 z-stacks of confocal images covering the full thickness of the pLLP were captured and presented as maximum projection in the figures. All of the time-lapse images were analyzed by the ImageJ software and then edited using the Adobe Photoshop® and Illustrator® software.

### Statistical analysis

All experiments were repeated at least three times and one representative result was shown. Data were presented as mean ± SEM. The number of embryos (*n* =) used in the experiments was indicated in each figure. A one-way analysis of variance (ANOVA) followed by Tukey test was used for multiple comparisons. A student's *t*-test was used for single comparisons.

## Result

### Lpar2b is expressed in the pLLP and NMs

**Figure 1:**
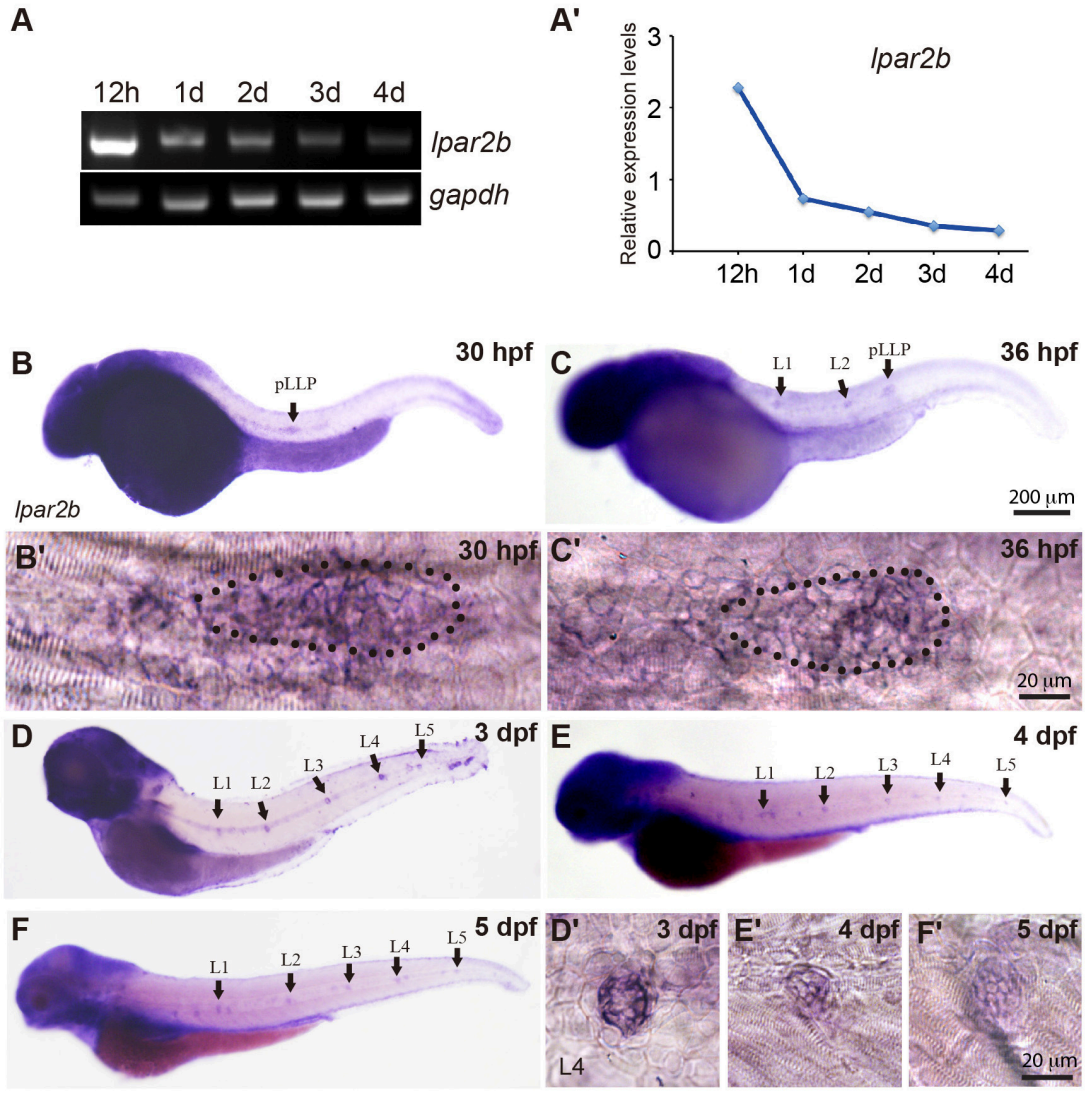
Lpar2b is expressed in the posterior lateral line in zebrafish. **(A)** RT-PCR shows the expression level of lpar2b in whole embryos from 12 hpf to 4 dpf. A house-keeping gene gapdh is used as control. **(A**′**)** Quantification of **(A)**. **(B–F)** Whole-mount ISH detects the transcripts of lpar2b in embryos from 30 to 5 dpf. **(B**′**,C**′**)** High-magnification images of the pLLP in **(B,C)**. The primordium is outlined by dots. **(D**′**-F**′**)** High-magnification images of the L4 NMs in **(D–F)**. Arrows, pLLP or NMs as indicated in the pictures. hpf, hours post fertilization; dpf, day post fertilization.

In a study investigating the expression pattern of LPA receptors, we found a LPA receptor Lpar2b expressed in the lateral line of zebrafish embryos. RT-PCR showed that during zebrafish development, the mRNA of Lpar2b was initially expressed at a relatively high level at 12 h post fertilization (12 hpf), and then gradually decreased from 1 to 4 dpf (Figures 1A,A′). Whole-mount *in situ* hybridization (ISH) using an anti-sense *lpar2b* RNA probe revealed that it was expressed in the migrating pLLP and the two deposited NMs (L1, L2) at 30 and 36 hpf (Figures 1B,C). Within the pLLP, Lpar2b appeared to be uniformly expressed (Figures 1B′,C′). At later stages, Lpar2b was continuously expressed in the NMs from 3 to 5 dpf (Figures 1D–F,D′-F′). These data indicate that Lpar2b may play an important role during lateral line development.

### Lpar2b is required for proper NM and hair cell development

To investigate the role of Lpar2b in lateral line development, we designed a translation-blocking Morpholino (MO) targeting the 5′-untranslated region (5′-UTR) of Lpar2b mRNA. Because there is no commercially available zebrafish Lpar2b antibody, we constructed a vector that infused the entire coding sequence of Lpar2b and part of its 5′UTR containing the MO-targeting site with GFP. The fusion Lpar2b-GFP mRNA was injected either alone or with the *lpar2b* MO into one-cell stage embryos and analyzed for green fluorescence at 8 hpf. Our results showed that the GFP fluorescence was strongly suppressed in the presence of *lpar2b* MO (Supplementary Figures 1A,B), indicating this MO can efficiently suppress the protein translation of Lpar2b mRNA *in vivo*.

**Figure 2:**
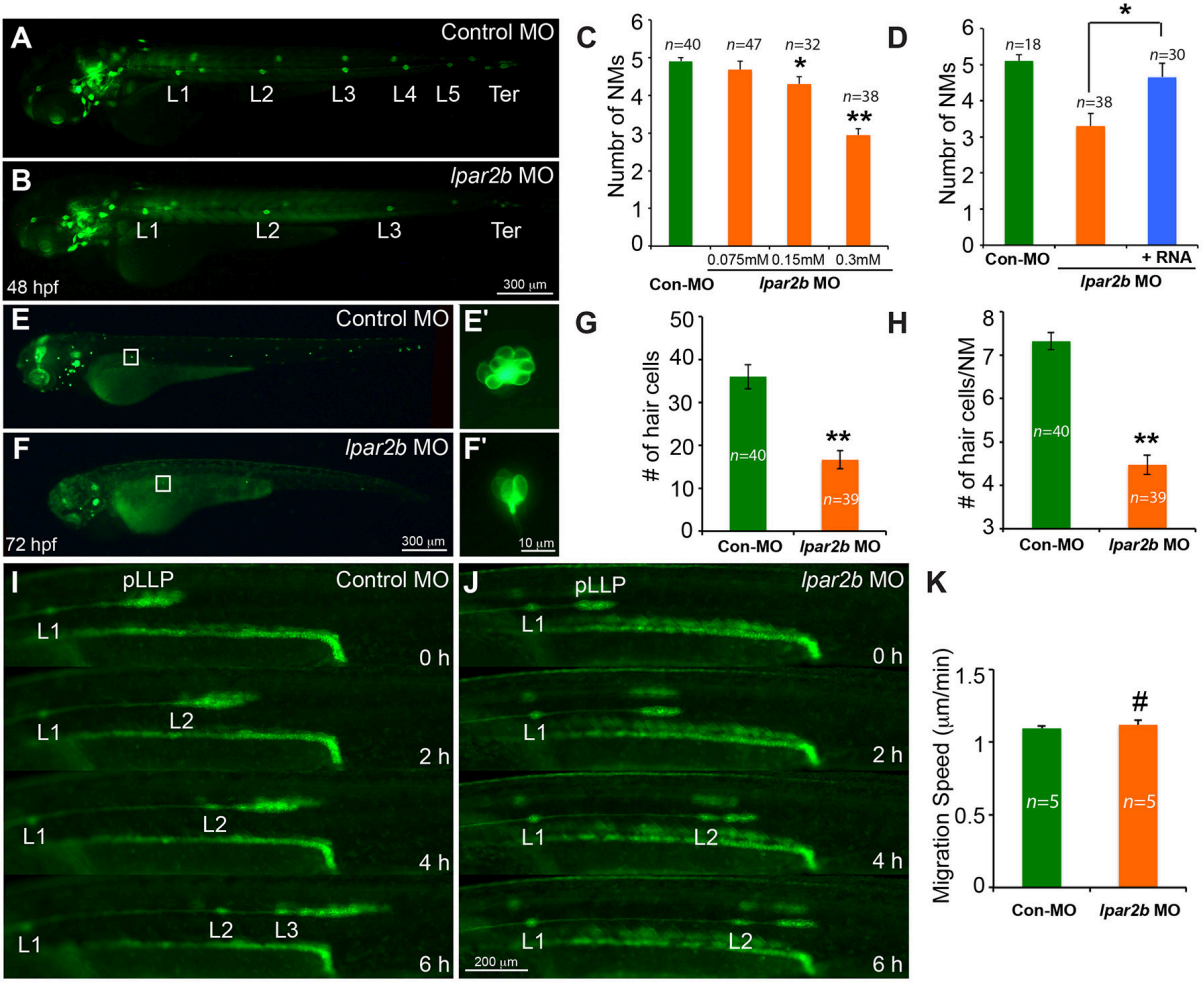
MO-mediated Lpar2b knock-down reduced the number of NMs and hair cells in the posterior lateral line. **(A,B)** Epifluorescence images of 48 hpf *Et(gata2:EGFP)*^*mp*189*b*^ embryos injected with control or lpar2b MO. **(C)** Quantification of the number of trunk NMs (exclude terminal NMs) in one side of the pLL in **(A,B)**. **(D)** Co-injection of MO-insensitive Lpar2b mRNA rescued the NMs phenotype caused by Lpar2b depletion. **(E,F)** Epifluorescence images of 72 hpf *Tg(Brn3c:mGFP)* embryos injected with control or lpar2b MO. **(E**′**,F**′**)** High-magnification images of hair cells in the L1 NMs in **(E,F)**. **(G)** Quantification of the total number of hair cells in one side of the pLL (L1-L5) at 72 hpf. **(H)** Average number of hair cells per NM (L1-L5) at 72 hpf. **(I,J)** Snapshots from 6-h epifluorescence time-lapses movies of control or lpar2b MO-injected *Tg(-8.0cldnb:lynEGFP)* embryos at 30–36 hpf (Supplementary Movies 1, 2). **(K)** Quantification of the migration speed of the pLLP of **(I,J)**. ^*^p < 0.05; ^**^p < 0.01; ^#^*p* > 0.05 compared to control.

We next injected the control or *lpar2b* MO into one-cell stage *Et(gata2:EGFP)*^*mp*189*b*^ zebrafish embryos. In this line, GFP is expressed in cells of the pLLP and NMs (Sang et al., 2014). By 48 hpf, the pLLP in control embryos had reached the tail region and the PLL now consisted of 5–6 trunk NMs (L1-L6) and 2–3 terminal NMs (Ter) on the tail (Figure 2A) (Xu et al., 2014). In Lpar2b morphants, regularly-spaced trunk and terminal NMs were visible at 48 hpf (Figure 2B), suggesting there was no major defect in pLLP migration. However, a dose-dependent reduction in the number of trunk NMs at 48 hpf was observed (Figures 2B,C). Specifically, the average number of trunk NMs was reduced to 2.95 ± 0.17 (*n* = 38) in embryos injected with 0.3 mM of *lpar2b* MO, significantly smaller compared to 4.9 ± 0.1 (*n* = 40) in control morphants (Figure 2C). To investigate whether Lpar2b also plays a role in hair cell development, we injected control or *lpar2b* MO into one-cell stage *Tg(Brn3c:mGFP)* embryos. In this line, hair cells of the inner ear and lateral line NMs express membrane-bound GFP (mGFP; Xiao et al., 2005). Fluorescence microscopy revealed that Lpar2b depletion significantly decreased the total number of hair cells in the trunk NMs, as well as the average number of hair cells per NM at 72 hpf (Figures 2E–H). To exclude the possibility of off-target effect of this MO, we designed a second *lpar2b* MO (referred to as *lpar2b* MO2) that blocks normal splicing of the *lpar2b* pre-mRNA. RT-PCR confirmed the efficacy of this MO (Supplementary Figure 1E) and its injection produced a similar NM phenotype to that of the first *lpar2b* MO (Supplementary Figures 1C–F). In addition, the NM phenotype in Lpar2b morphants can be rescued by co-injection of a MO-insensitive *lpar2b* mRNA (Figure 2D). To better corroborate the *lpar2b* MO effects, we generated genetic mutations of the *lpar2b* gene by the CRISR-Cas9 system in the background of *Et(gata2:EGFP)*^*mp*189*b*^. A guide-RNA (gRNA) was designed against the 5' region of the 3^rd^ exon of zebrafish *lpar2b* gene (Supplementary Figure 2A) and its injection with the Cas9 mRNA into one-cell stage embryos produced indels in the predicted region with high efficacy (Supplementary Figure 2B). Importantly, injection of the *lpar2b* gRNA and Cas9 mRNA produced a same NM phenotype in a large proportion of F0 embryos (Supplementary Figures 2C,D) as that of *lpar2b* MO. Together, these results indicate the observed NM phenotype in *lpar2b* morphants was caused specifically by Lpar2b depletion.

To better understand the cellular mechanism underlying this NM phenotype, we performed *in vivo* time-lapse imaging of the pLLP on the *Tg(-8.0cldnb:lynEGFP)* fish. In this transgenic line, membrane-bound EGFP was specifically expressed in cells of the pLLP and NMs (Haas and Gilmour, 2006). Time-lapse imaging showed that the pLLP migration was normal in Lpar2b morphants compared to control (Figures 2I,J; Supplementary Movies 1, 2). The migration speed of pLLP was also comparable between the two groups (Figure 2K). However, it took longer time for the pLLP depleted of Lpar2b to deposit NMs (Figures 2I,J). During the 6-h time-lapse imaging, control pLLP deposited two NMs (L2, L3) along the path of migration, while the pLLP of the Lpar2b morphants only deposited one (Figures 2I,J; Supplementary Movies 1, 2). These results indicate that the NM phenotype in Lpar2b morphants was caused by a slower rate of NM deposition.

### Lpar2b depletion disrupts the normal patterning of the pLLP

**Figure 3:**
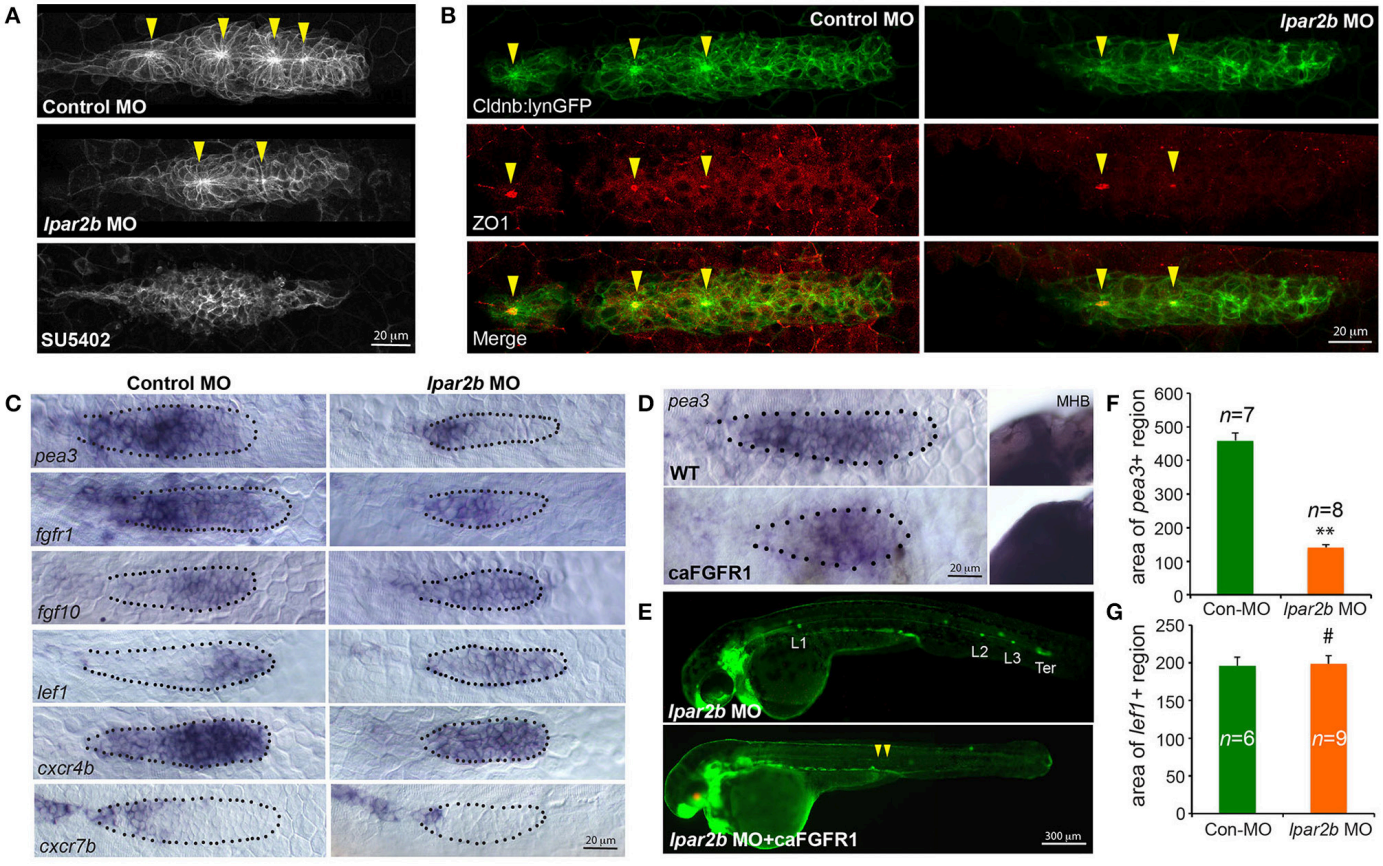
Lpar2b regulates the patterning of the pLLP. **(A)** Confocal images showing the rosette structures in the pLLP of *Tg(-8.0cldnb:lynEGFP)* embryos at 32 hpf. **(B)** Immunostaining of ZO1 (red) in the pLLP of *Tg(-8.0cldnb:lynEGFP)* embryos at 32 hpf. Arrowheads mark the center of rosettes in **(A,B)**. **(C)** Whole-mount ISH shows the expression of genes as indicated in the pLLP at 30–32 hpf in embryos injected with control or lpar2b MO. **(D)** Whole-mount ISH detects the expression of pea3 in the pLLP and midbrain-hindbrain boundary (MHB) regions in wild-type (WT) and *Tg(hsp70:caFGFR1)* embryos at 32 hpf. The *Tg(hsp70:caFGFR1)* embryos were heat shocked at 24 hpf for 1 h at 37°C. The pLLP is outlined by dots in **(C,D)**. **(E)** Epifluorescence images showing *Tg(-8.0cldnb:lynEGFP) /Tg(hsp70:caFGFR1)* double transgenic embryos injected with lpar2b MO. Upper panel, without heat shock; lower panel, with heat shock. Arrowheads, a trail of cells left by pLLP. **(F,G)** Quantification of the area expressing pea3 **(F)** or lef1 **(G)** in the pLLP. ^**^p < 0.01; ^#^*p* > 0.05 compared to control.

FGF signaling drives the formation of apical constriction and rosettes (pro-NMs) in the trailing region of pLLP (Lecaudey et al., 2008; Nechiporuk and Raible, 2008). To investigate if the observed NM phenotype in Lpar2b morphants was caused by abnormal FGF activity, we first examined the rosette structure in the pLLP in *Tg(-8.0cldnb:lynEGFP)* embryos. Confocal microscopy showed that the pLLP in Lpar2b morphants appeared smaller than that of control (Figures 3A,B). While control pLLP normally contained 3–4 rosettes at 30–32 hpf, pLLP depleted of Lpar2b only had 1–2 rosettes at this time (Figure 3A, arrowheads). Immunofluorescence staining with an epithelialization marker ZO1 that labels the center of rosettes showed a similar result (Figure 3B), suggesting a reduced FGF signaling in the pLLP. We next performed whole-mount ISH to determine the FGF activity in the primordium. Consistent with the confocal result, pLLP depleted of Lpar2b appeared smaller compared to control (Figure 3C). In the control primordium, an FGF ligand *fgf10* was expressed in the leading 1/3 region, and an FGF receptor *fgfr1* and a downstream reporter *pea3* were expressed in the trailing 3/4 of the pLLP (Figure 3C, left panels) (Lecaudey et al., 2008). In Lpar2b morphants, the expression of *fgfr1* appeared normal in the pLLP (Figure 3C, right panel). However, the *pea3* domain was greatly reduced to the trailing 1/4 of the pLLP, and the *fgf10* region was expanded to almost the whole primordium (Figure 3C, right panels). Quantification of the area of *pea3* showed this area in Lpar2b morphants is only 30.5 ± 2.2% of that of control (Figure 3F). These results indicate a smaller FGF active region and a disrupted patterning in the pLLP depleted of Lpar2b.

Since FGF and Wnt signaling play antagonizing roles in pLLP patterning (Thomas et al., 2015), we reason that the observed reduction in FGF signaling may be caused by an increased Wnt activity in the leading region. A Wnt effector Lef1 is normally expressed in the leading 1/4 region of pLLP (Figure 3C, left panel; Aman and Piotrowski, 2008). In the pLLP of Lpar2b morphants, the *lef1*+ domain was relatively expanded to the leading 3/4 region (Figure 3C, right panel). Interestingly, the absolute area of *lef1*+ region in Lpar2b morphants was comparable to that of control (Figure 3G), suggesting the relative expansion of Wnt into the trailing part was caused by an overall smaller pLLP size. It has been shown that a chemokine receptor Cxcr4b was expressed in the leading 3/4 region, and Cxcr7b was expressed in the trailing edge in a complement pattern (Figure 3C, left panel; Aman and Piotrowski, 2008). In the *Apc*^*mcr*^ mutant, constitutive activation and expansion of the Wnt/β-catenin signaling leads to expansion of *cxcr4b* and loss of *cxcr7b* in the pLLP (Aman and Piotrowski, 2008). We therefore ask if the relative expansion of Wnt in Lpar2b morphants would lead to similar changes in *cxcr4b* and *cxcr7b*. Indeed, ISH showed that Lpar2b depletion caused an expansion of *cxcr4b* to the entire primordium and a reduction of *cxcr7b* in the trailing edge (Figure 3C). These data supports the idea that Lpar2b depletion caused a relative expansion of Wnt in the pLLP.

We next investigated if transgenic expression of a constitutively-active Fgfr1(caFGFR1) is able to rescue the NM phenotype in Lpar2b morphants. For this purpose, we crossed the *Tg(hsp70:caFGFR1)* line to the *Tg(cldnb:lynEGFP)* background, and heat-shocked these double transgenic embryos at 24 hpf and monitored their lateral line development. Expression of a heat-shock induced red fluorescence marker in the lens and an expansion of the *pea3*+ domains in the pLLP and other regions (Figures 3D,E) confirmed the induction of the caFGFR1 transgene. Surprisingly, activation of FGF signaling failed to rescue the NM defect in Lpar2b morphants but instead produced a very different phenotype (Figure 3E). In these Lpar2b-depleted embryos with elevated FGF activity, the pLLP terminated its migration prematurely, leaving a narrow trail of cells along the myoseptum at 48 hpf (Figure 3E, arrowheads). On the other hand, inhibition of the FGF signaling by SU5402 completely suppressed rosette formation (Figure 3A) and blocked normal pLLP migration (data not shown) (Lecaudey et al., 2008), which is also different from Lpar2b knock-down. Taken together, these results suggest the reduced FGF signaling in the pLLP is unlikely primarily responsible for the NM phenotype. Instead, the smaller FGF region in Lpar2b morphants is likely due to a relatively expanded Wnt region caused by the reduction in overall pLLP size.

### Lpar2b regulates the tissue size of the pLLP

**Figure 4:**
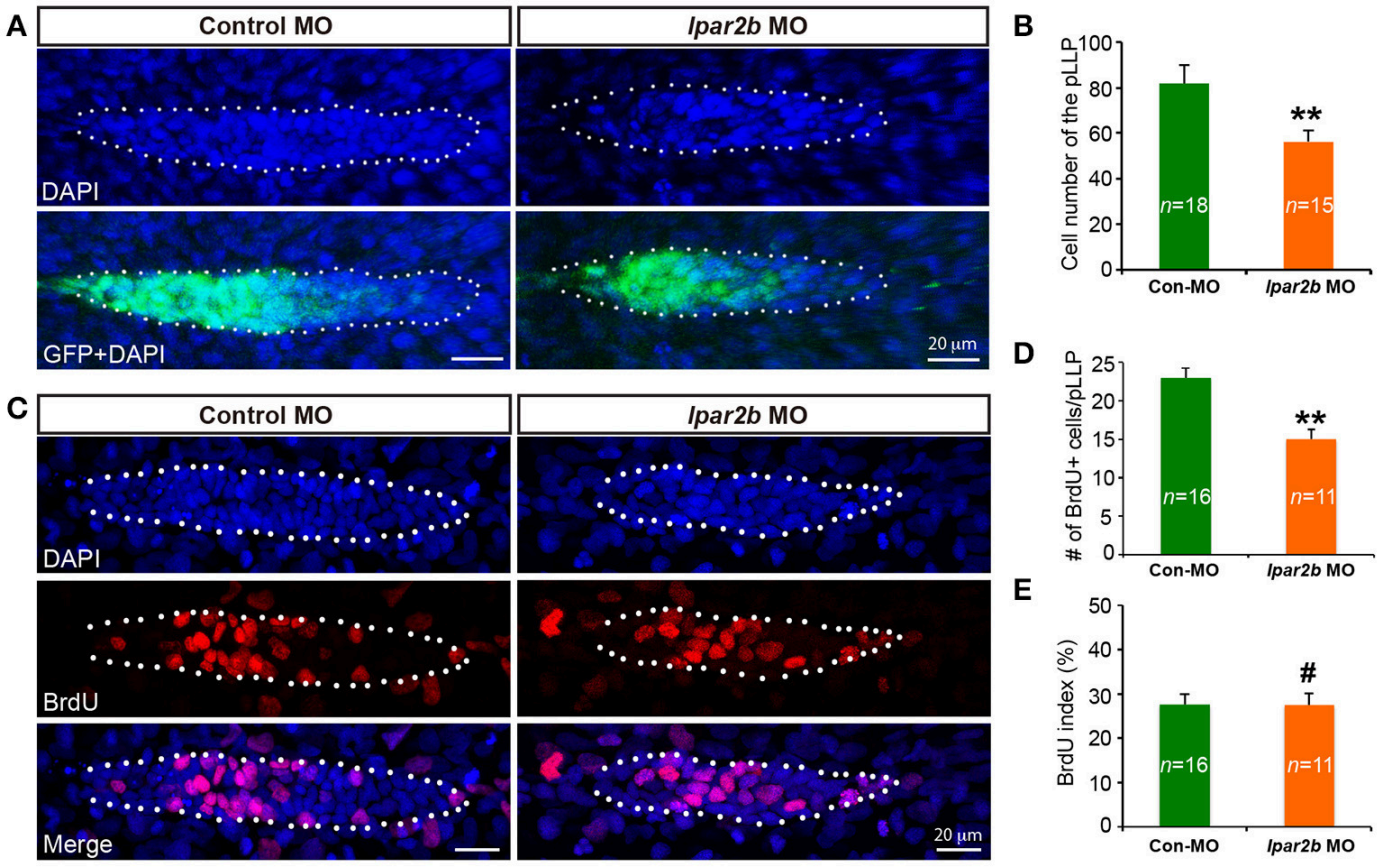
Lpar2b regulates the tissue size of the pLLP. **(A)** Confocal images showing the pLLP of control- or lpar2b MO-injected *Et(gata2:EGFP)*^*mp*189*b*^ embryos at 32 hpf. The embryos were stained with DAPI to show cell nuclei (blue). GFP comes from the gata2:EGFP transgene. The pLLP is outlined by dots. **(B)** Quantification of the total number of cells in the pLLP. **(C)** Confocal images of the pLLP at 32 hpf after DAPI (blue) and BrdU (red) staining. **(D)** Quantification of the number of BrdU+ cells in the pLLP. **(E)** The BrdU index is calculated as (number of BrdU+ cells in pLLP)/(total number of pLLP cells). Con-MO, Control MO; ^**^p < 0.01; ^#^*p* > 0.05 compared to control.

It has been previously shown that the cell number of pLLP affects the rate of NM deposition (Aman et al., 2011). Reduced number of pLLP cells caused by either decreased proliferation or increased apoptosis leads to fewer deposited NMs (Aman et al., 2011), similar to the NM phenotype observed in Lpar2b morphants. As the pLLP appears smaller in Lpar2b morphants than that of control, we asked whether Lpar2b regulates its tissue size. Indeed, confocal microscopy showed that the total number of pLLP cells decreased significantly in Lpar2b morphants compared to control at 32 hpf (Figures 4A,B). Specifically, control pLLP contains an average of 82.00 ± 7.96 cells (*n* = 18), while pLLP depleted of Lpar2b only have 56.87 ± 7.05 cells (*n* = 15), which is about 2/3 of control (Figure 4B). As cell number is often controlled by cell proliferation and apoptosis, we first examined cell proliferation in the primordium by performing a BrdU assay that detects cells in the S phase of the cell cycle. BrdU immunofluorescence showed a significant decrease in BrdU+ cells in pLLP depleted of Lpar2b (Figures 4C,D). Specifically, control primordium contains an average of 23 ± 5.11 BrdU+ cells (*n* = 16), while pLLP depleted of Lpar2b only have 15 ± 4.31 BrdU+ cells (*n* = 11) at 32 hpf. Interestingly, the BrdU index of the pLLP determined by the ratio of the number of BrdU+ nuclei to the total number of nuclei, is comparable between the two groups at this time point (Figure 4E). We next questioned whether the smaller pLLP is a result of increased apoptosis in Lpar2b morphants. TUNEL assay showed no difference in cell apoptosis between control and Lpar2b morphants (Supplementary Figure 3). In both groups, rarely any TUNEL+ cells can be found within the pLLP (Supplementary Figure 3), suggesting the reduced pLLP size is independent of apoptosis. Together, these data indicate Lpar2b regulates the tissue size of the pLLP.

### Lpar2b controls pLLP size and NM number by regulating Yap1 phosphorylation

**Figure 5:**
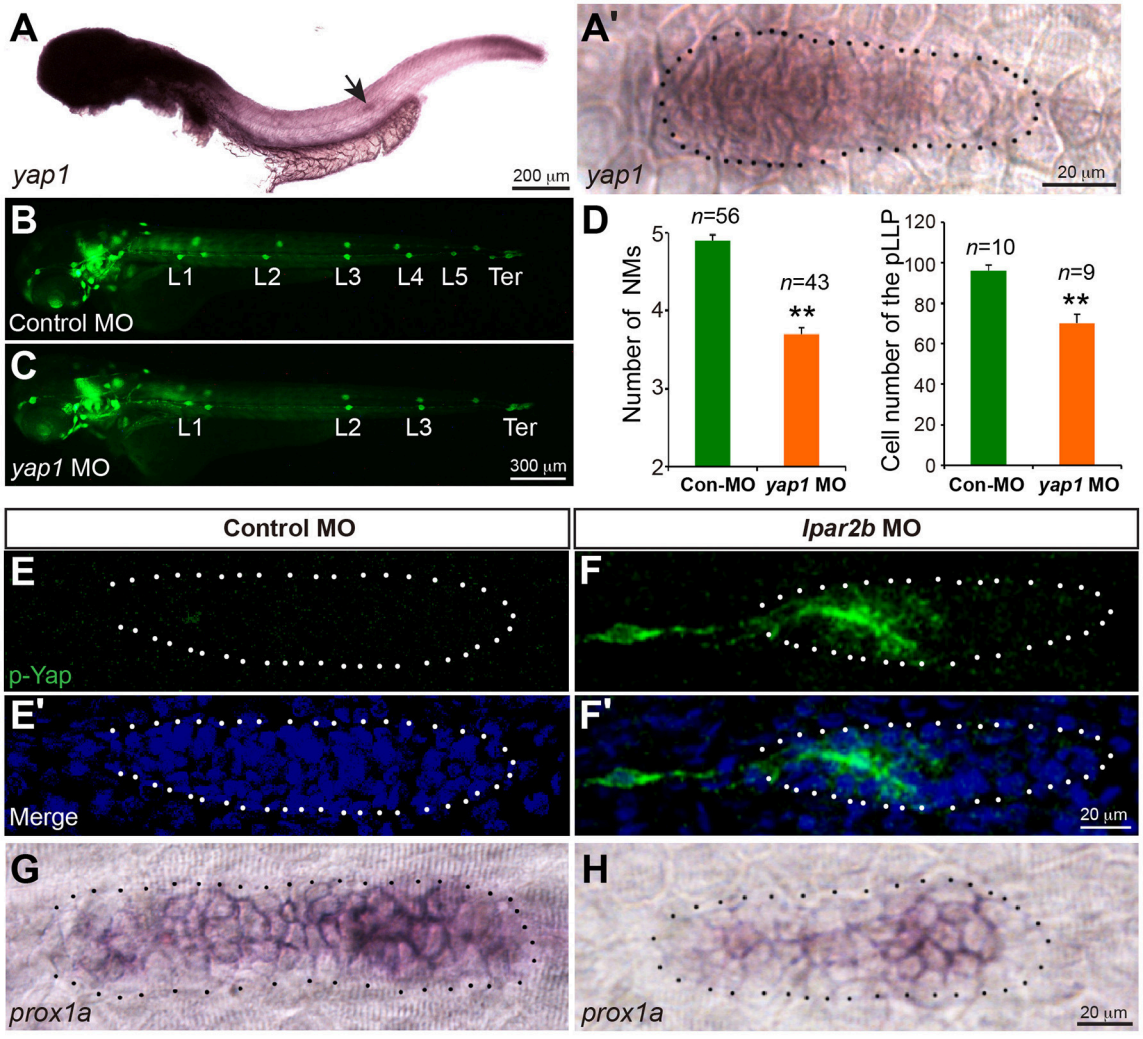
Lpar2b regulates Yap1 activity. **(A,A**′**)** Whole-mount ISH detects the mRNA of yap1 in embryos at 34 hpf. **(A**′**)** High-magnification of the pLLP in **(A)**. **(B,C)** Epifluorescence images of control- or yap1 MO-injected *Et(gata2:EGFP)*^*mp*189*b*^ embryos at 48 hpf. **(D)** Quantification of the NM number at 48 hpf and number of pLLP cells at 32 hpf in control- or *yap1* MO-injected embryos. **(E–F**′**)** Confocal images of the pLLP stained with DAPI (blue) and p-Yap1 (green) at 32 hpf. **(G,H)** ISH shows a decreased *prox1a* expression in the pLLP of Lpar2b morphants. The pLLP is outlined by dots. ^**^p < 0.01 compared to control.

The Hippo pathway play important roles in organ size control (Yu et al., 2015). It was recently shown that a key Hippo pathway effector *yap1* is expressed in the pLLP (Kozlovskaja-Gumbriene et al., 2017). In addition, this pathway is known to be the downstream of LPA receptors in cell culture (Yu et al., 2012). We therefore first performed ISH to confirm the expression of Yap1 in the pLLP. ISH showed that the *yap1* mRNA is enriched in the migrating pLLP (Figures 5A,A'), consistent with the previous report (Kozlovskaja-Gumbriene et al., 2017). Interestingly, knocking-down Yap1 with a previously validated translation-blocking MO (He et al., 2015) significantly reduced the number of cells in the pLLP, as well as the number of deposited NMs, phenocopying the result of Lpar2b knock-down (Figures 5B–D). This suggests Yap1 may be the downstream of Lpar2b in the pLLP.

We next ask whether Lpar2b regulates Yap1 activity in the primordium. A whole-mount immunofluorescence staining was performed against p-Yap1, the inactive form of Yap1 (Yu and Guan, 2013). Though the p-Yap1 antibody was synthesized with a peptide corresponding to residues surrounding Ser127 of human p-Yap1, the residues of this region are almost identical between zebrafish and human (Supplementary Figure 4A). Immunostaining with this antibody produced strong cytoplasmic signals in pronephric duct cells of control embryos (Supplementary Figure 4B), consistent with a previous report (He et al., 2015). Importantly, knocking down the endogenous Yap1 with the Yap1 MO almost completely abolished the signal (Supplementary Figure 4B), indicating the p-Yap1 signal is specific. We then examined the expression of p-Yap1 in the pLLP. In control embryos, p-Yap1 was expressed at a very low level in the trailing part of the pLLP (Figures 5E,E'). In contrast, Lpar2b knock-down resulted a much higher p-Yap1 expression (Figures 5F,F'). In these embryos, strong p-Yap1 signal was present in the trailing half of the primordium (Figures 5F,F', Supplementary Figure 4C), and co-injection of the Yap1 MO abolished the signal (Supplementary Figure 4C). Furthermore, Lpar2b knock-down suppressed the expression of *prox1a*, a downstream target gene of Yap1 in the pLLP (Figures 5G,H; Loh et al., 2014). These results indicate Lpar2b suppresses Yap1 phosphorylation and thus increases its activity in the pLLP.

**Figure 6:**
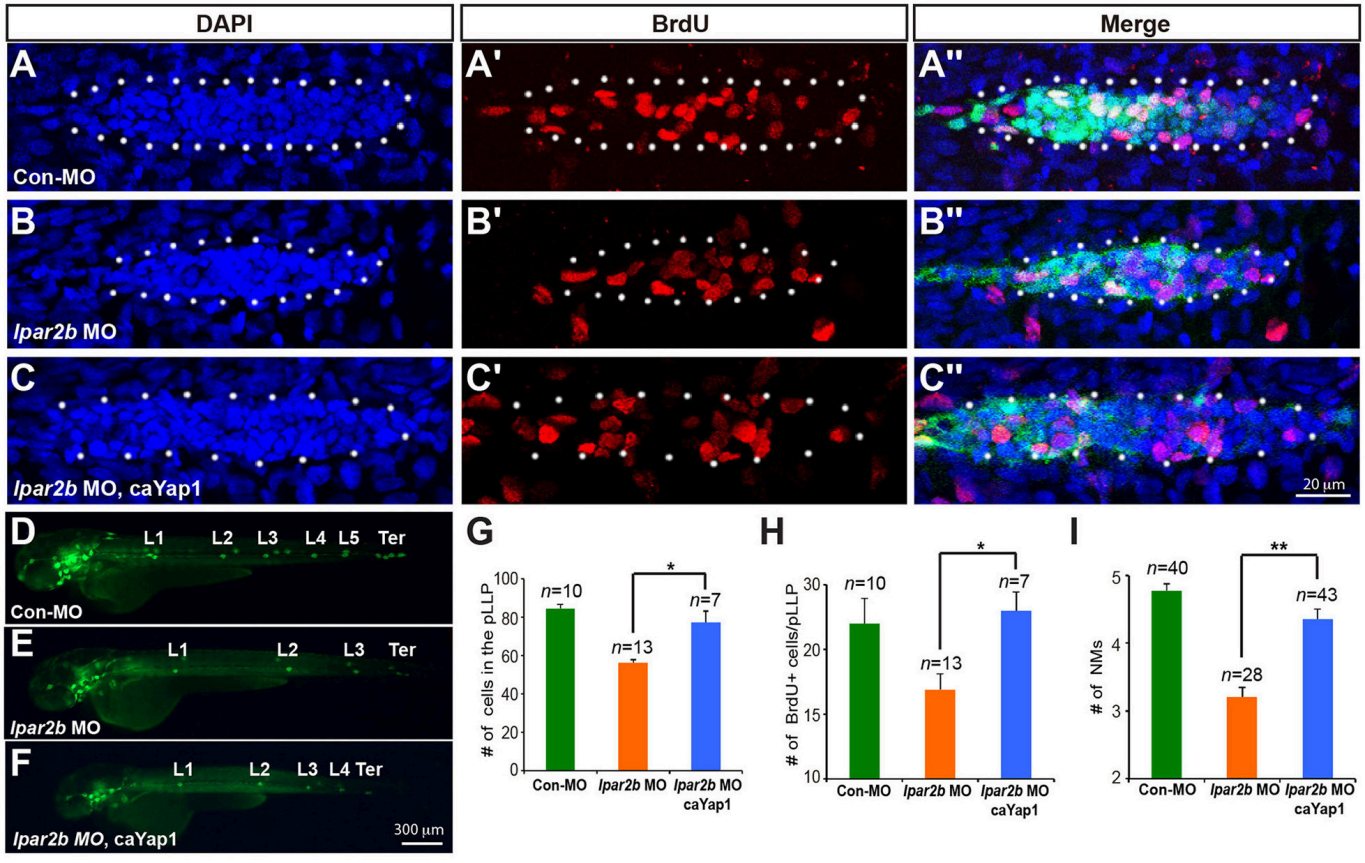
A phosphorylation-resistant Yap1 mRNA (caYap1) rescued the pLLP and NM phenotype of Lpar2b morphants. **(A–C**″**)** Confocal images of the pLLP stained with DAPI (blue) and BrdU (red) at 32 hpf in *Et(gata2:EGFP)*^*mp*189*b*^ embryos injected with control MO **(A–A**″**)**, lpar2b MO **(B–B**″**)** or *lpar2b* MO and caYap1 mRNA together **(C–C**″**)**. The pLLP is outlined by dots. **(D–F)** Epifluorescence image s of *Et(gata2:EGFP)*^*mp*189*b*^ embryos injected with control MO **(D)**, *lpar2b* MO **(E)**, or *lpar2b* MO and caYap1 mRNA together **(F)** at 48 hpf. **(G–I)** Quantification of the total number of cells **(G)** and the number of BrdU+ cells **(H)** in the pLLP at 32 hpf, and the number of NMs at 48 hpf (I) in 3 groups of embryos as indicated. ^*^p < 0.05; ^**^p < 0.01.

To investigate whether Lpar2b regulates the pLLP tissue size and NM number through Yap1, we constructed a mutated Yap1 (referred to as caYap1) that is resistant to phosphorylation and thus constitutively active (Zhao et al., 2007). Strikingly, injection of the caYap1 mRNA rescued the pLLP and NMs phenotype caused by Lpar2b knock-down (Figures 6A–F). Specifically, the caYap1 mRNA recovered the number of pLLP cells (Figures 6A–C,G, from 56.31 ± 1.56 to 77.43 ± 5.85, *p* < 0.05), as well as the number of proliferating cells in the pLLP of Lpar2b morphants (Figures 6A′-C′,H, from 16.91 ± 1.21 to 23 ± 1.45, *p* < 0.05). Expression of the caYap1 also significantly increased the number of deposited NMs in Lpar2b depleted embryos at 2 dpf (Figures 6D–F,I, from 3.21 ± 0.14 to 4.36 ± 0.15, *p* < 0.01). Taken together, our results indicate Lpar2b controls pLLP size and NM number by regulating Yap1 activity.

## Discussion

LPA signaling influences the migration, proliferation, survival, differentiation, and adhesion of many cell types during physiological and pathological conditions (Okudaira et al., 2010). Previous studies have shown that many genes in the LPA signaling pathway are highly conserved among vertebrates (Nakanaga et al., 2010), suggesting the importance of their functions. In this study, we investigated the expression and function of a LPA receptor Lpar2b in PLL development. Whole-mount ISH revealed that Lpar2b is expressed in the pLLP and NMs, and loss-of-function studies showed that this LPA receptor is required for the proper development of NMs and hair cells. In addition, immunofluorescence and ISH showed Lpar2b is required for normal pLLP patterning. Further analyses indicate Lpar2b controls pLLP size and NM number by regulating Yap1 activity. Our study thus uncovered a novel role of LPA signaling in organ size control in the lateral line, and may have important implications of its potential role in the development of other organs.

The G-protein coupled LPA receptor LPA2 plays important roles during cell migration. CD4+ T cells in mice lacking LPA2 (*Lpar2*^−/−^) display a severe defect in cell motility during the early homing process at the lymph nodes (Knowlden et al., 2014). LPA2 is also required for gradient sensing during the chemotaxis of fibroblasts (Ren et al., 2014). In an *in vivo* model of collective cell migration, LPA2 induces cell-cell dissociation and promotes a partial epithelial-mesenchymal transition (EMT) in *Xenopus* neural crest (NC) cells by down-regulating cell-cell adhesions (Kuriyama et al., 2014). It's therefore surprising that we didn't observe any significant defect in pLLP migration in Lpar2b morphants. Since other LPA receptors (Lpar2a, Lpar3-6) may also be expressed in the pLLP, it's possible that they have redundant functions and the depletion of only Lpar2b is insufficient to cause a migration phenotype. It has been shown that the chemokine receptors Cxcr4b and Cxcr7b are expressed in a complement manner and control the directional migration of pLLP by self-generating a Cxcl12a gradient (Donà et al., 2013; Venkiteswaran et al., 2013). In Lpar2b morphants, the Cxcr4b domain is expanded and the Cxcr7b region is greatly reduced (Figure 3C). Interestingly, time-lapse imaging shows that the migration of pLLP is normal (Figures 2I–K, Supplementary Movies 1, 2), suggesting even a small amount of Cxcr7b expressed in the trailing edge is sufficient to establish a functional Cxcl12a gradient across a migrating tissue.

**Figure 7:**
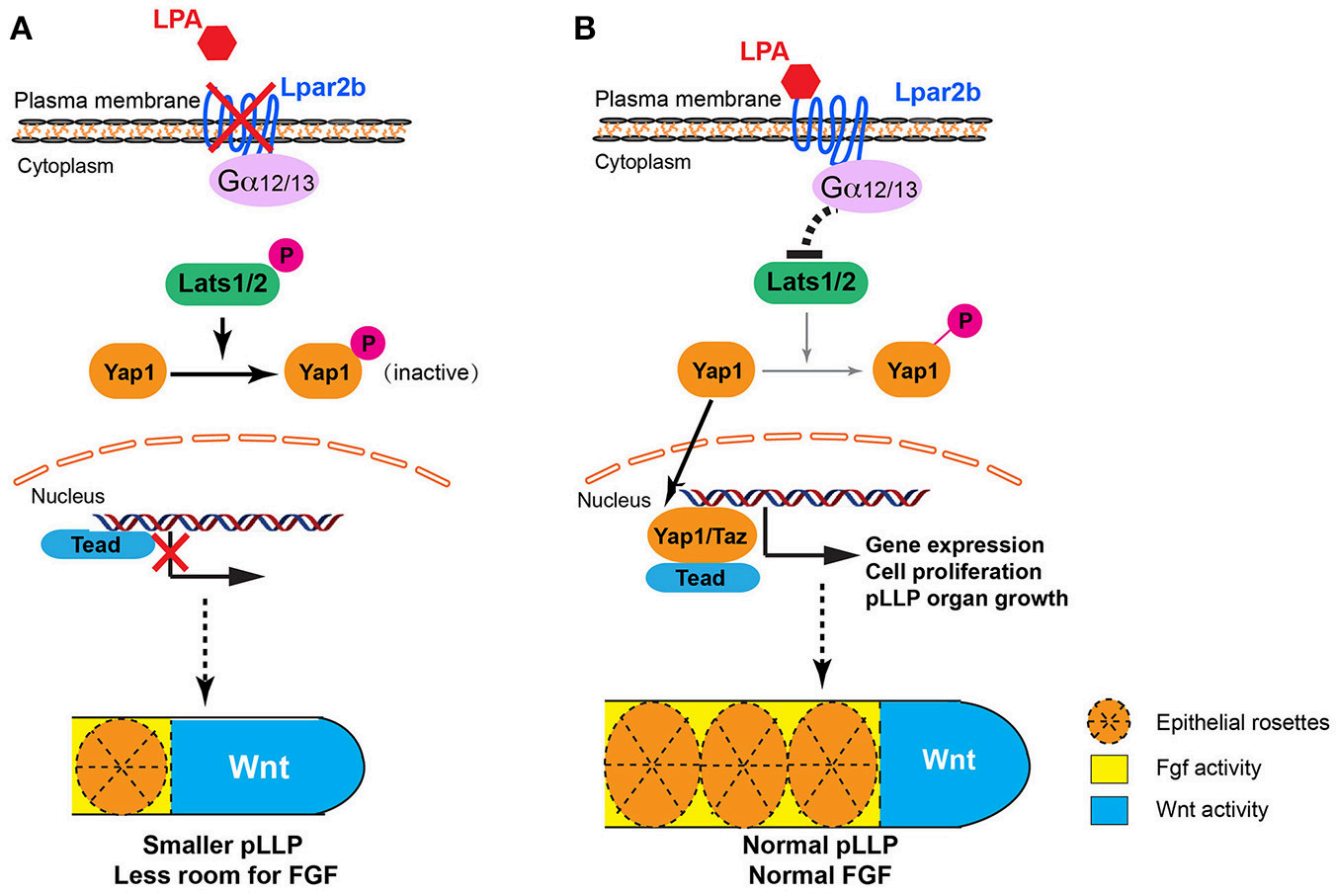
Proposed models for LPA signaling in tissue growth and patterning of the pLLP. **(A)** When Lpar2b is depleted, Lats1/2 is active and phosphorylates Yap1. P-Yap1 is retained in the cytoplasm and becomes inactive. Genes required for cell proliferation and organ growth is not expressed, resulting in a smaller pLLP size. As the Wnt domain (blue) remains constant, the FGF region (yellow) is thus more restricted to the posterior edge, which in turn reduces the number of rosettes that can be formed within the pLLP. **(B)** When Lpar2b is present in the pLLP, it's activated upon LPA binding, and presumably signals through its coupled G-proteins to inhibit Lats1/2 activity. Yap1 is thus not phosphorylated and translocates to the nucleus to activate gene transcription that promotes growth of the pLLP. The pLLP reaches its normal size, which provides enough space for FGF signaling and rosette formation.

Previous studies revealed that antagonizing Wnt and FGF signaling controls the patterning of the pLLP (Dalle Nogare and Chitnis, 2017). Wnt induces the expression of FGF ligands Fgf3/10a in the leading region, whereas FGF inhibitors Sef and Dusp6 induced by Wnt restricts the FGF signaling to the trailing edge (Aman and Piotrowski, 2008; Matsuda et al., 2013). FGF regulates the formation of epithelial rosettes in the trailing region and also induces the expression of a Wnt inhibitor Dkk1b, which in turn suppresses the activation of Wnt in the trailing edge (Aman and Piotrowski, 2008). In our study, Lpar2b depletion resulted in a reduced number of NMs and rosettes in the primordium, and further ISH analysis showed a reduced FGF region and a comparable Wnt domain (in absolute size) in the pLLP (Figure 3C). We initially speculated that the NM phenotype was caused by an inhibition of the FGF signaling in the pLLP. However, further analyses indicate there is no direct interaction of LPA/Hippo signaling with the FGF pathway in the pLLP, based on several results: (1) Although the *pea3* region is reduced in Lpar2b morphants, it's always expressed in the posterior edge, which is different from SU5402 treatment that completely abolishes *pea3* expression in the primordium (Aman and Piotrowski, 2008). (2) An FGF inhibitor SU5402 completely inhibited the formation of epithelia rosettes (Figure 3A), as well as pLLP migration (data not shown; Lecaudey et al., 2008). While in Lpar2b morphants, rosettes are still formed regularly (though fewer in number), and the pLLP migration is normal. (3) If FGF is the downstream of Lpar2b, one would expect activating FGF generates more NMs and a larger pLLP. However, activating FGF with hsp70:caFGFR1 greatly suppressed pLLP cell proliferation (Aman et al., 2011), and consistently we found the pLLP with elevated FGF became smaller and eventually disappeared (Figure 3D and not shown). Together, our data suggest the change of FGF region after Lpar2b knockdown is indirect and secondary, likely caused by a decreased pLLP size. We therefore propose a model to illustrate how LPA/Hippo regulates pLLP tissue growth, and how Lpar2b depletion changes pLLP patterning (Figure 7). We propose that as the total cell number decreased significantly while the Wnt active region remains constant in Lpar2b morphants, there is less room left for FGF signaling in the primordium (Figure 7A). As a result, fewer rosettes can be formed within the FGF region, and *pea3* expression is further restricted to the trailing edge (Figures 3, 7A).

In this study, we show that Lpar2b controls the tissue size of pLLP by regulating the activity of a key Hippo effector Yap1 *in vivo*. Based on our knowledge, this study is the first report showing LPA signaling regulates organ size through the Hippo pathway during vertebrate development. The exact cellular mechanism underlying this organ size control by Lpar2b is still unclear and requires further investigation. We observed a reduced number of BrdU+ cells in the pLLP of Lpar2b morphants, while the BrdU index is normal at 32 hpf. It is likely that compensatory mechanisms were activated after Lpar2b knock-down to maintain a certain number of cells and the general structure of pLLP. Because the BrdU only labels cells at the S phase, it's also possible that pLLP cells still have cell-cycle defects in other phases such as G1 or M that is not shown by BrdU labeling. In addition, we can't exclude the possibility that Lpar2b regulates pLLP cell number through a proliferation-independent mechanism. Since our study is performed on an *in vivo* model, it's worthwhile to further investigate how Lpar2b regulates Yap1 activity during lateral line development. Specifically, which G-protein is required and whether Lpar2b regulates Yap1 through Mst1/2, which was previously shown not regulated by LPA in cell culture system (Yu et al., 2012). In our study, we also found that Lpar2b depletion significantly reduced the number of hair cells in the lateral line (Figures 2E–H). Since Lpar2b is expressed in the NMs from 3 to 5 dpf (Figures 1D–F), it will be interesting to investigate how LPA regulates hair cell development, and whether it's through the Hippo pathway. As the Hippo pathway are required for organ and tissue regeneration (Wang et al., 2017), it will also be interesting to study if LPA signaling plays a role during hair cell regeneration.

## Author contributions

XW and HH: performed the experiments; XW, HH, KS, ZZ, SZ, YC, LC, QS, FL, and HX: analyzed the data; QS, FL, and HX: designed the experiments; HX: wrote the paper.

### Conflict of interest statement

The authors declare that the research was conducted in the absence of any commercial or financial relationships that could be construed as a potential conflict of interest.
