# Supplementary material for: Lpar2b Controls Lateral Line Tissue Size by Regulating Yap1 Activity in Zebrafish
Source: Front Mol Neurosci. 2018 Feb 9;11:34. doi: 10.3389/fnmol.2018.00034 (PMC5812253; doi:10.3389/fnmol.2018.00034)
Supplement: Supplementary file 3 [file DataSheet1.pdf]

Supplementary Figure and Movie legends

Figure S1. Validation of the efficacy of the MOs used in the study. (A-B) Fluorescence images showing 8-hpf embryos injected with the Lpar2bGFP construct (A) alone, or together with *lpar2b* MO (B). (C,D) Epifluorescence images of control MO- or *lpar2b* MO2-injected *Et(gata2:EGFP)*^{mp189b} embryos at 48 hpf. (E) The splicing-blocking *lpar2b* MO2 disrupts normal slicing of the *lpar2b* pre-mRNA as shown by RT-PCR. (F) Quantification of the number of NMs at 48 hpf in (C,D). *, $p < 0.05$ compared to control.

Figure S2. Genetic mutation of *lpar2b* generated by the CRISPR-Cas9 system produces a similar NM phenotype in F0 embryos. (A) The gRNA was designed against the 5' region of exon 3 of *lpar2b* genomic DNA. (B) Sequencing of the genomic DNA extracted from F0 embryos injected with the gRNA and Cas9 mRNA. (C) Fluorescence images of control *Et(gata2:EGFP)*^{mp189b} embryos injected with Cas9 mRNA alone, and embryos injected with *lpar2b* gRNA and Cas9 together at 48 hpf. (D) Quantification of the percentage of embryos with normal pLLP migration but fewer NMs (< 5 trunk NMs) at 48 hpf.

Figure S3. Lpar2b knock-down does not increase cell apoptosis in the pLLP. (A-H) Confocal images showing the pLLP after TUNEL staining (red) in control- (A-D) or *lpar2b* MO-injected (E-H)*Tg(-8.0cldnb:lynEGFP)* embryos at 32 hpf. Cell nuclei were stained with DAPI (blue). Rarely any TUNEL+ cell (red) can be observed in the pLLP in both groups.

Figure S4. Validation of the p-Yap1 antibody used in the study. (A) Alignment of the N-terminal amino acid sequences of human (hsYAP1) and zebrafish Yap1 (drYap1) proteins. High concensus sequences (>90%) are shown in red, and low concensus sequences (>50%) are depicted in blue. Arrow, the Ser127 phosphorylation site recognized by the antibody. (B)Confocal images of the pronephric duct region stained with p-Yap1 and DAPI in 32 hpf embryo injected with control- of *yap1* MO. Images in the left 2 panels are generated from maximum projection of 6 confocal z-stacks. A single z-stack of the boxed areas is

depicted in the right 3 panels. Note the p-Yap1 signal is in the cytoplasm and does not overlap with the nucleus. (C) Confocal images of the pLLP cells stained with p-Yap1 and DAPI in embryos injected with indicated MOs. The p-Yap1 signal is in the cytosol of pLLP cells.

Movie 1. Migration of the pLLP in control embryos. *Tg(-8.0cldnb:lynEGFP)* embryos were injected with control MO. Time-lapse experiments were performed at 30-36.5 hpf. Fluorescence images were captured at 4-minute intervals, using a Leica DMI6000 inverted microscope with a 5× objective. The movie plays at 7 frames/sec.

Movie 2. Migration of the pLLP in Lpar2b-depleted embryos. *Tg(-8.0cldnb:lynEGFP)* embryos were injected with *lpar2b* MO. Time-lapse experiments were performed at 30-36.5 hpf. Fluorescence images were captured at 4-minute intervals, using a Leica DMI6000 inverted microscope with a 5× objective. The movie plays at 7 frames/sec.

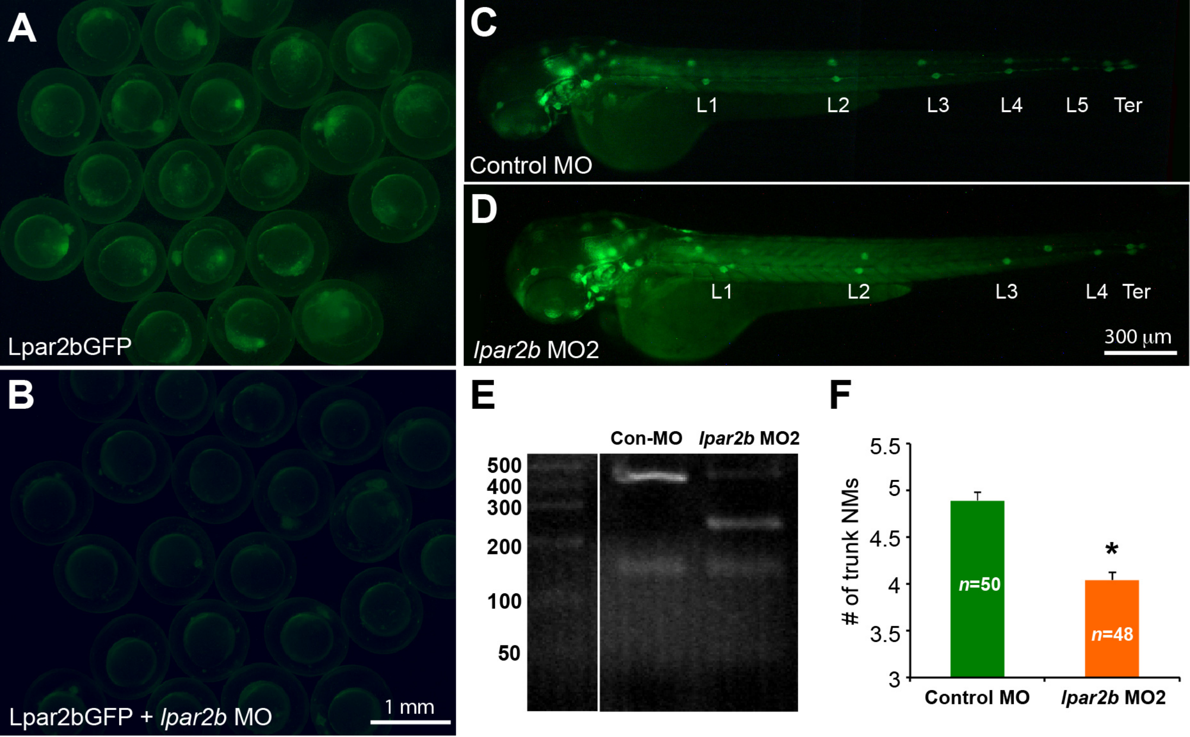

Supplementary Figure S1

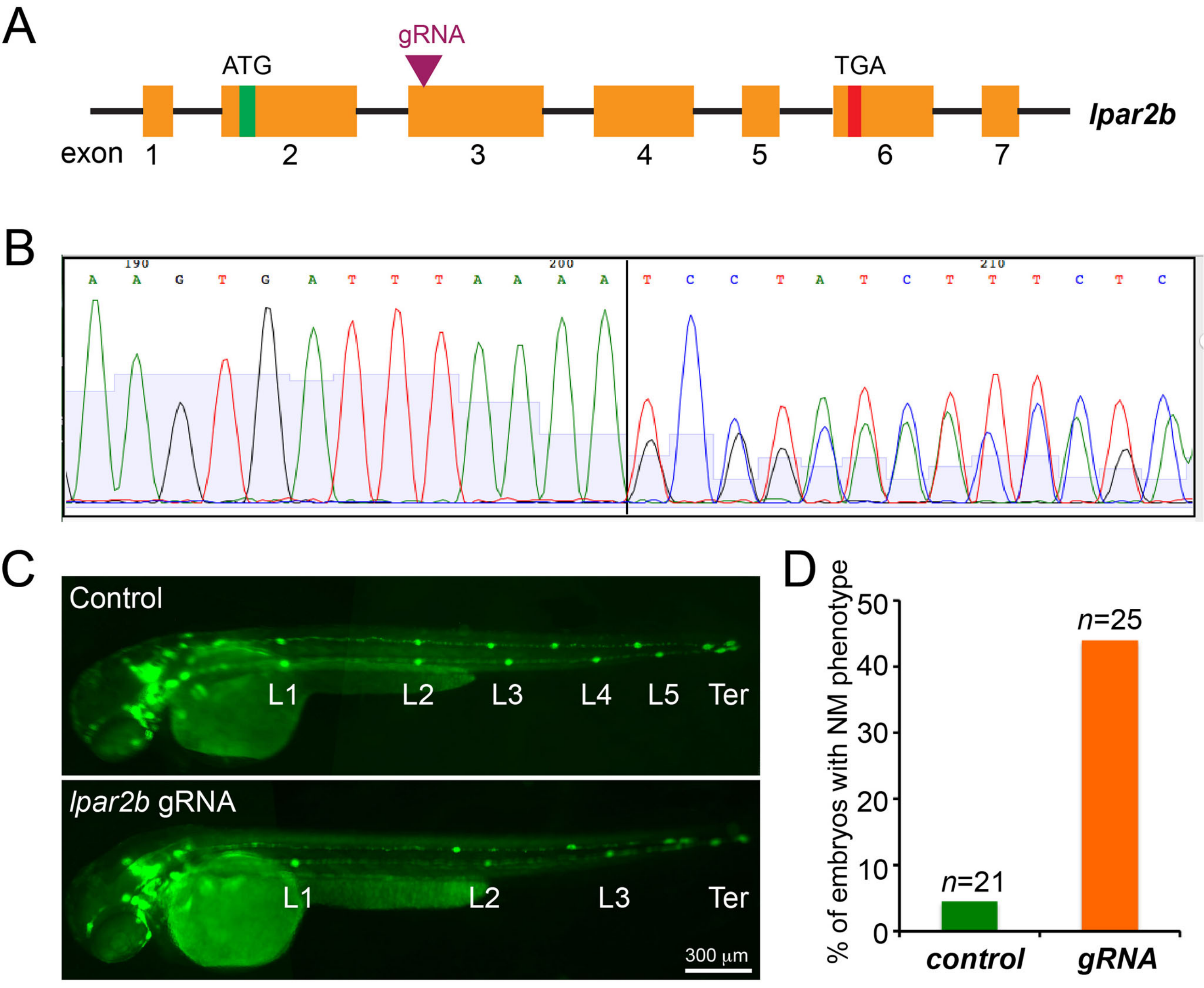

supplementary Fig. S2

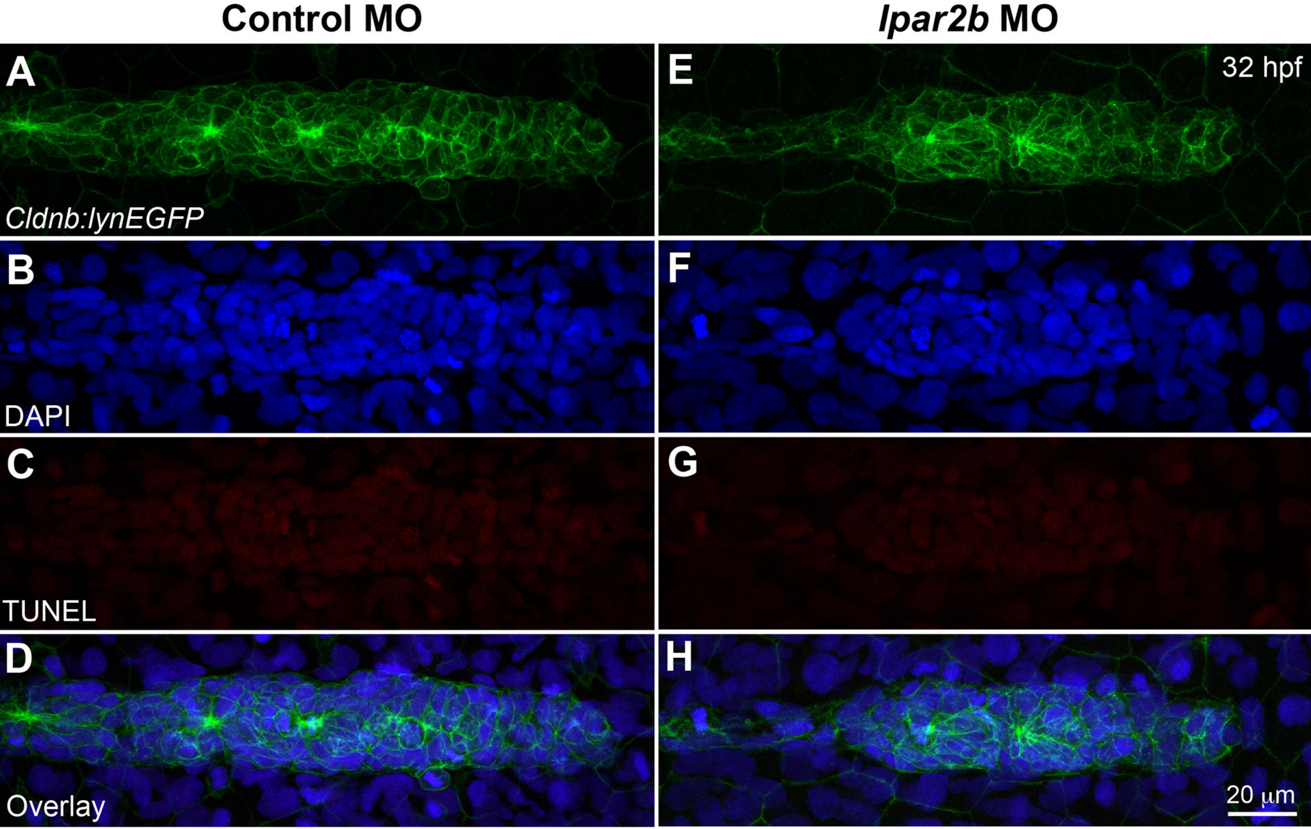

Supplementary Figure S3

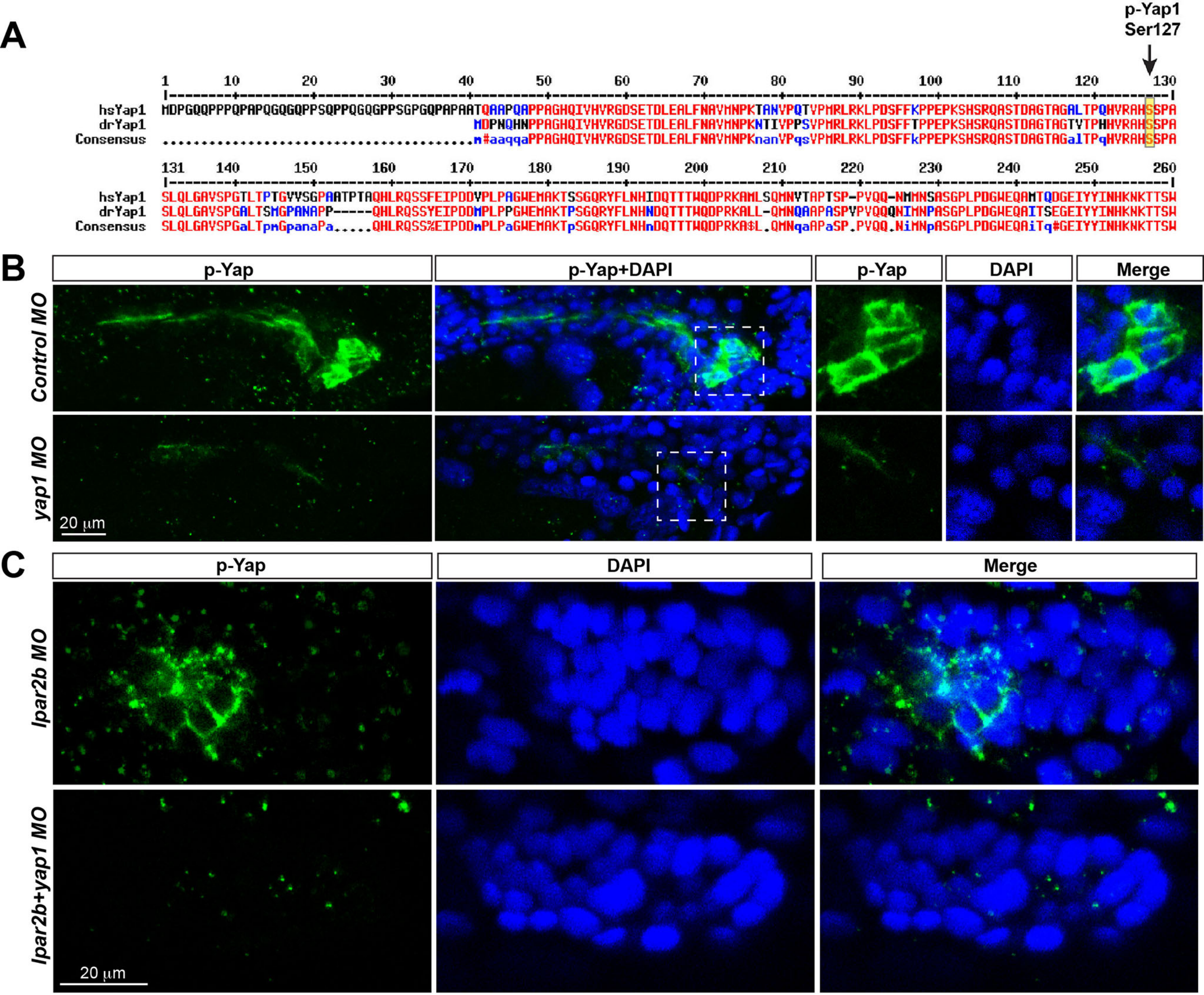

Supplementary Figure S4
